# Stat3 Activation Is Limiting for Reprogramming to Ground State Pluripotency

**DOI:** 10.1016/j.stem.2010.06.022

**Published:** 2010-09-03

**Authors:** Jian Yang, Anouk L. van Oosten, Thorold W. Theunissen, Ge Guo, Jose C.R. Silva, Austin Smith

**Affiliations:** 1 Wellcome Trust Centre for Stem Cell Research & Department of Biochemistry, University of Cambridge, Tennis Court Road, Cambridge, CB2 1QR, UK

**Keywords:** STEMCELL

## Abstract

The cytokine leukemia inhibitory factor (Lif) sustains self-renewal of mouse embryonic and induced pluripotent stem cells by activating Jak kinase and the transcription factor Stat3. Here we investigate whether Jak/Stat3 may also contribute to induction of pluripotency. EpiSCs derived from postimplantation embryos express low levels of Lif receptor and Stat3. We introduced into EpiSCs a Jak/Stat3 activating receptor (GY118F) responsive to granulocyte colony stimulating factor (Gcsf). On transfer to ground state culture, in which MAPK signaling and glycogen synthase kinase are inhibited, Gcsf induced transcriptional resetting and functional reprogramming. Activation of a tamoxifen-regulatable fusion, Stat3ER^T2^, also converted EpiSCs into chimera-competent iPSCs. We exploited GY118F to increase Jak/Stat3 activity during somatic cell reprogramming. Incompletely reprogrammed cells derived from neural stem cells or fibroblasts responded to Gcsf with elevated frequencies of progression to ground state pluripotency. These findings indicate that Jak/Stat3 participate directly in molecular reprogramming and that activation of this pathway is a limiting component.

## Highlights

► LIF promotes induction of pluripotency independent of its role in self-renewal ► Increased Jak/Stat3 activation is sufficient to convert EpiSCs to naive pluripotency ► Jak/Stat3 activation is a limiting factor in somatic cell reprogramming ► Jak/Stat3 facilitates progression of intermediate states to pluripotency

## Introduction

Rodent embryonic stem cell (ESC) lines are derived from the pluripotent epiblast of preimplantation embryos (Batlle-Morera et al., 2008; Brook and Gardner, 1997; Evans and Kaufman, 1981; Martin, 1981). They retain the unique capacity of naive pluripotent cells to colonize blastocyst chimeras and contribute to all somatic lineages plus the germline (Bradley et al., 1984). A characteristic feature of both mouse and rat ESCs is responsiveness to leukemia inhibitory factor (Lif) (Buehr et al., 2008; Smith et al., 1988; Williams et al., 1988). Lif stimulates self-renewal via the gp130 signal transducing receptor that activates Jak kinases and thence the transcriptional mediator Stat3 (Matsuda et al., 1999; Niwa et al., 1998).

Heterogeneous cell lines, termed EpiSCs, can be established by culture of postimplantation epiblast (Brons et al., 2007; Tesar et al., 2007). EpiSCs have capacity for multilineage differentiation in the context of teratoma formation but they do not reproducibly colonize chimeras. Unlike ESCs, EpiSCs do not utilize the Lif/Stat3 pathway and instead rely on activin and fibroblast growth factor (Fgf). EpiSCs differ from ESCs in various other respects. They do not express or have substantially downregulated several transcription factor markers of ESCs and early epiblast. Conversely they show activated expression of germ layer specification markers such as brachyury (T). Accordingly, we have suggested that EpiSCs may represent a primed state of pluripotency that is poised for lineage commitment (Nichols and Smith, 2009). Indeed, ongoing differentiation is a routine feature of EpiSC cultures. Furthermore, EpiSCs are epigenetically distinct from ESCs. In female ESCs, as in early epiblast, both X chromosomes are active. Female EpiSCs, however, display the histone modification signature of X inactivation (Guo et al., 2009).

ESCs can be differentiated into EpiSCs by withdrawal of Lif and culture in activin and Fgf without feeders (Guo et al., 2009). It has recently been reported that EpiSCs maintained on feeders in the presence of serum-derived components may produce ESC-like cells (Bao et al., 2009; Greber et al., 2010). This is suggested to represent dedifferentiation. However, it has also been shown that EpiSCs on feeders continuously produce primordial germ cell precursors that may then undergo epigenetic conversion into pluripotent embryonal germ (EG) cells (Hayashi and Surani, 2009).

Importantly for our study, EpiSCs maintained without feeders or serum factors spontaneously generate ES-like cells at a frequency less than 1 in 10^6^ (Guo et al., 2009; Hanna et al., 2009). However, they convert at frequencies of 0.1%–1.0% by transcription factor directed reprogramming mediated by Klf4, Klf2, or Nanog (Guo et al., 2009; Hall et al., 2009; Hanna et al., 2009; Silva et al., 2009). This reprogramming is completely suppressed if cells are maintained in activin and Fgf, demonstrating the dominant influence of extrinsic cues. Conversion into EpiSC-derived induced pluripotent stem cells (Epi-iPSCs) only proceeds on withdrawal of these factors and transfer into ground state ESC culture conditions (Ying et al., 2008). This comprises serum-free medium containing two selective small molecules (2i) that respectively inhibit Fgf stimulation of mitogen-activated protein kinases Erk1 and Erk2 and constitutive activity of glycogen synthase kinase-3 (Gsk3). The ground state conditions are also effective in promoting incompletely reprogrammed somatic cells to pluripotent status (Silva et al., 2008; Sridharan et al., 2009). Notably, although 2i can sustain ESC self-renewal without Stat3 activation (Ying et al., 2008), Lif is routinely added to this and other mouse reprogramming media. Here we investigated whether the contribution of Lif and downstream activation of Stat3 is limited to maximizing the self-renewal of iPSCs or might include an active role in the reprogramming process.

## Results

### Lif Increases the Efficiency of EpiSC Reprogramming

**Figure 1:**
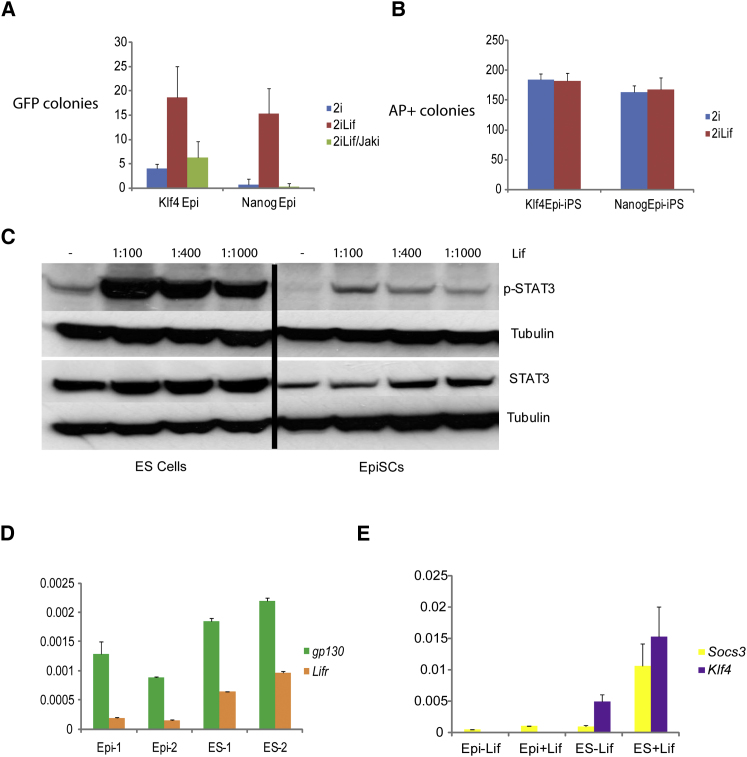
Lif Enhances EpiSC Reprogramming (A) Yield of Oct4-GFP-positive Epi-iPSC colonies from Klf4- or Nanog-transfected EpiSCs transferred to 2i, 2i plus Lif, or 2i plus Lif with JAK inhibitor. (B) Colony formation by Epi-iPSCs in 2i or 2i/Lif. (C) Immunoblot analysis of Stat3 and phosphoStat3 in ESCs and EpiSCs. Cells were cultured in unsupplemented medium for 4 hr, then stimulated with indicated concentrations of Lif for 20 min. (D) qRT-PCR analysis of *gp130* and *Lifr* expression in EpiSCs or ESCs. (E) qRT-PCR analysis of *Socs3* and *Klf4* expression in EpiSCs or ESCs with or without Lif stimulation for 1 hr. Data on *Tbx3* expression are presented in Figure S1. Error bars are standard deviations from the mean of triplicate determinations.

We compared the frequency of Epi-iPSC generation in the presence or absence of Lif. We used embryo-derived Oct4-GFP (O4G) reporter EpiSCs stably transfected with expression constructs for Klf4 or Nanog (Guo et al., 2009). On transfer from activin plus Fgf into 2i with Lif, these cells produced GFP-positive iPSC colonies at a frequency of 0.5%–1% (Guo et al., 2009; Silva et al., 2009). Without Lif or in the presence of a Jak inhibitor, this yield was reduced several fold (Figure 1A). To test whether the effect of Lif is due simply to increased efficiency of iPSC self-renewal, we plated reprogrammed Epi-iPSCs in 2i with or without Lif. We observed no significant difference in numbers of colonies formed or their undifferentiated phenotype (Figure 1B).

These observations suggested that Lif responsiveness might be limiting for reprogramming to the pluripotent ground state. We examined Stat3 expression and activation in EpiSCs. Compared with ESCs, EpiSCs express less Stat3 protein and show much reduced phosphorylation of tyrosine 705 in response to Lif (Figure 1C). Weak activation may also be partly attributable to lower expression of the Lif receptor (Figure 1D). Interrogation of two direct Stat3 targets, *Socs3* and *Klf4* (Hall et al., 2009), confirmed diminished Lif/Stat3 signal transduction in EpiSCs (Figure 1E). Interestingly, *Socs3* is weakly induced whereas *Klf4* shows no response. Klf4 is a reprogramming factor (Takahashi and Yamanaka, 2006) and also a mediator of ESC self-renewal (Niwa et al., 2009). EpiSCs also fail to express *Tbx3* (Figure S1 available online), which has been implicated in ESC self-renewal downstream of PI3 kinase (Niwa et al., 2009).

### Increased Activation of Stat3 Drives EpiSC Reprogramming

**Figure 2:**
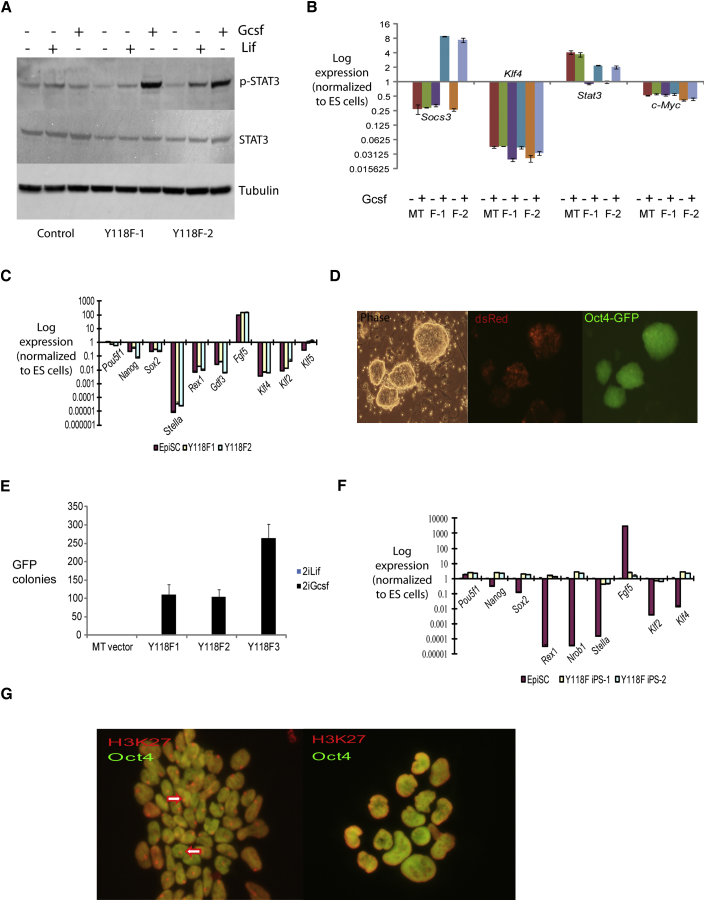
Reprogramming of EpiSCs Transfected with GY118F (A) Western blot analysis of p-Stat3 induction in parental or GY118F EpiSC clones after Lif or Gcsf stimulation for 20 min. (B) qRT-PCR analysis of empty vector (MT) and two clones of GY118F EpiSCs stimulated with Gcsf for 1 hr. (C) Marker gene profile of parental and GY118F EpiSCs cultured in activin/Fgf2. Expression levels are relative to *Gapdh* and normalized to undifferentiated ESCs. (D) Oct4-GFP-positive colonies obtained from GY118F EpiSCs transferred to 2i/Gcsf. dsRed fluorescence indicates transgene expression. Images were taken after 8 days in 2i/Gcsf. (E) Number of Oct4-GFP-positive colonies after culturing three different clones of GY118F EpiSCs in 2i/Gcsf for 8 days. Data on colony yield in Gcsf alone are presented in Figure S2. (F) qRT-PCR analysis of marker gene expression in GY118F Epi-iPSC clones relative to *Gapdh* and normalized to ESCs. (G) H3K27me3 staining of female O4G EpiSCs (left) showing nuclear foci (arrowed) and derivative GY118F Epi-iPSCs reprogrammed in 2i/Gcsf (right) with no evident foci. Characterization of Epi-iPSCs after tatCre-mediated excision of the GY118F transgene is presented in Figure S3. Error bars are standard deviations from the mean of triplicate determinations.

We engineered EpiSCs to express a chimeric receptor, GY118F, that elicits hyperactivation of endogenous Jak and Stat3 (Niwa et al., 1998). In this receptor the ligand binding domain of the granulocyte colony stimulating factor (Gcsf) receptor is fused to the transmembrane and cytoplasmic domains of the Lif receptor signal transducer gp130. The gp130 cytoplasmic domain is modified by conversion of residue 118 from tyrosine to phenylalanine. This abolishes the docking site for Shp2 which couples to Ras-Mapk and PI3 kinase. Thus GY118F signals only via Jak and Stat3 (Burdon et al., 1999a). Furthermore, because tyrosine 118 is also the binding site for the negative feedback regulator Socs3 (Schmitz et al., 2000), GY118F induces elevated and sustained activation of Jak/Stat3 (Burdon et al., 1999b). We generated O4G EpiSC clones stably expressing GY118F by plasmid transfection or *PB* transposition. Equivalent results were obtained with both types of transfectant. Parental EpiSCs do not express Gcsf receptor and show no response to Gcsf (data not shown). In GY118F transfectants exposed to Gcsf, Stat3 was tyrosine phosphorylated (Figure 2A) and Socs3 was induced to levels even higher than in ESCs (Figure 2B). Stat3 is autoregulatory and its expression increased around 2-fold. Strikingly, however, there was no induction of Klf4, nor of cMyc. No phenotypic change or alteration in ESC markers was apparent in cells maintained in activin plus Fgf (Figure 2C). We transferred clonal GY118F transfectants into 2i with Lif or Gcsf. In 2i/Lif, cells died or differentiated and no colonies were recovered. In contrast, 100–300 Oct4-GFP-positive colonies were obtained per plate of 2 × 10^4^ cells transferred into 2i plus Gcsf (Figures 2D and 2E). We also obtained colonies with Gcsf alone without addition of the two inhibitors, albeit at much lower frequency (Figure S2). Nanog-GFP reporter EpiSCs transfected with GY118F and cultured in 2i/Gcsf produced numerous colonies with upregulated Nanog-GFP (data not shown). Substantiating reprogramming, expanded O4G Epi-iPSCs showed the marker signature of naive pluripotency with loss of EpiSC features (Figure 2F; Figure S3). Epigenetic resetting was evidenced by loss of the H3K27me3 nuclear body diagnostic of a silenced X chromosome (Figure 2G). We used tatCre protein transduction (Peitz et al., 2002) to excise the floxed transgene from PB-generated Epi-iPSCs. Deletion was monitored by loss of dsRed fluorescence and confirmed by genomic PCR and RT-PCR (Figure S3). Cre-excised cells retained the ground state marker profile establishing that the reprogrammed state is stable. Functional pluripotency was confirmed by generation of multiple chimeras before and after Cre excision (Table 1).

**Figure 3:**
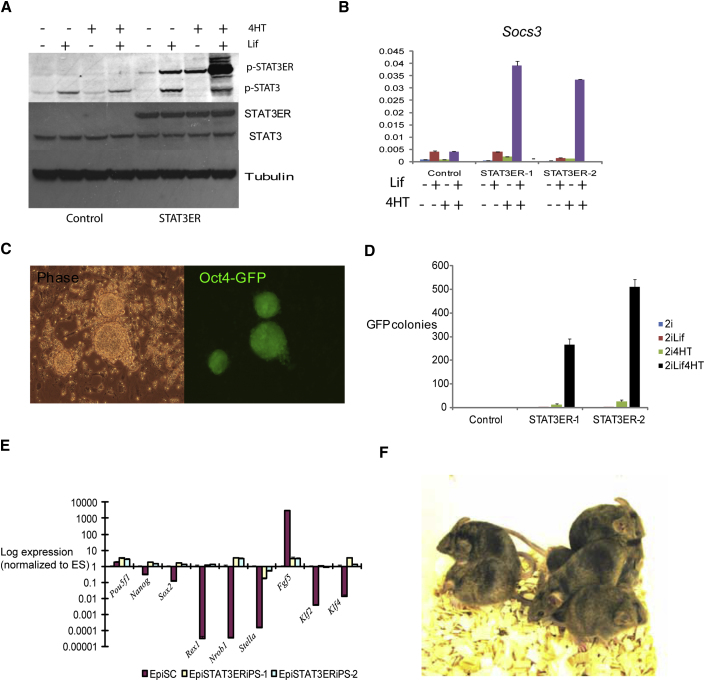
Reprogramming EpiSCs by Tamoxifen Activation of Stat3ER^T2^ (A) Immunoblot analysis of Stat3 and Stat3ER^T2^ tyrosine phosphorylation in EpiSC transfectants exposed to 4-hydroxytamoxifen (4HT) or Lif for 1 hr. (B) qRT-PCR analysis of *Socs3* induction by 4HT and Lif for 1 hr. (C) Oct4-GFP colonies obtained from Stat3ER^T2^ EpiSCs after 8 days in 2i/Lif plus 4HT. (D) Mean numbers of Oct4-GFP colonies from triplicate wells of Stat3ER^T2^ EpiSCs after 8 days in 2i with or without Lif and/or 4HT. (E) qRT-PCR analysis of marker gene expression in Stat3ER^T2^ Epi-iPSC clones relative to *Gapdh* and normalized to ESCs. (F) Chimeric mice produced from Stat3ER^T2^ Epi-iPSCs. Error bars are standard deviations from the mean of triplicate determinations.

To test further whether Stat3 mediates EpiSC reprogramming, we used a hormone regulatable Stat3ER^T2^ fusion (Bourillot et al., 2009). Tamoxifen-induced activation of Stat3ER can replace Lif in ESC maintenance (Bourillot et al., 2009; Matsuda et al., 1999). We introduced Stat3ER^T2^ into O4G EpiSCs. Stable transfectants showed tamoxifen-dependent accumulation of phosphorylated Stat3ER^T2^ and induction of Socs3 (Figures 3A and 3B). Provision of Lif with tamoxifen further activated Stat3ER^T2^ and increased Socs3 induction to similar levels as in GY118F transfectants. Klf4 was not induced, however, and the cells retained EpiSC identity. On transfer to 2i with tamoxifen plus Lif, they generated Epi-iPSCs with an efficiency of 1%–2% (Figures 3C–3E). Only few colonies were generated with tamoxifen alone, further indicating that the amount of Stat3 activated is critical. It is also possible that Jak signaling makes a contribution independently of Stat3 by direct modification of chromatin (Dawson et al., 2009). Cells reprogrammed with tamoxifen plus Lif were injected into blastocysts and gave good contribution to adult chimeras (Figure 3F, Table 1).

### Jak/Stat3 Functions Early and Transiently in EpiSC Reprogramming

**Figure 4:**
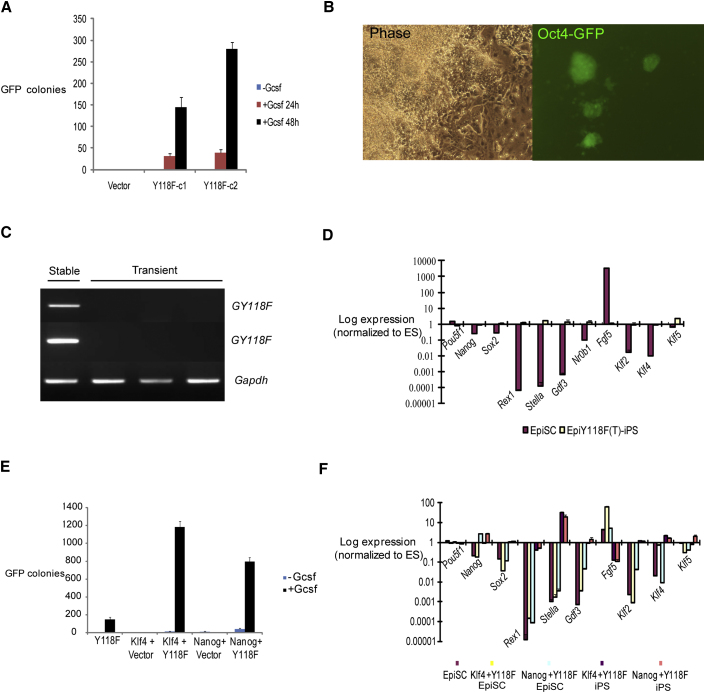
Jak/Stat3 Acts Early in Reprogramming and Synergizes with Klf4 and Nanog (A) Number of Oct4-GFP colonies from 2 × 10^4^ GY118F EpiSCs cultured in triplicate in 2i/Gcsf 24 hr or 48 hr then switched to 2i/Lif and scored at day 8. (B) Oct4-GFP colonies obtained by transient transfection with GY118F and culture in 2i/Gcsf for 8 days. (C) Genomic PCR (top) and RT-PCR (middle and bottom) analyses of Epi-iPSCs generated by stable or transient transfection. (D) Marker profile of Epi-iPSCs derived from EpiSCs by transient expression of GY118F. (E) Number of Oct4-GFP colonies generated by EpiSCs stably transfected with GY118F and either Klf4 or Nanog then transferred to 2i/Gcsf for 8 days. (F) qRT-PCR analysis of marker gene expression relative to *Gapdh* and normalized to ESCs for GY118F+Klf4 and GY118F+Nanog double transfected EpiSCs and derivative iPSC clones. Error bars are standard deviations from the mean of triplicate determinations.

To assess the timeframe of Jak/Stat3 action during EpiSC reprogramming, we added Gcsf to GY118F transfectants for only the initial 24 or 48 hr after transfer to 2i. Cultures were subsequently maintained in 2i plus Lif. Epi-iPSC colonies were obtained after 24 hr stimulation and the yield after 48 hr was similar to that obtained with continuous stimulation (Figure 4A). This prompted us to assess transient transfection with GY118F. After lipofection with circular GY118F vector, O4G EpiSCs were transferred into 2i with either Lif or Gcsf. With Gcsf we obtained between 5 and 32 Oct4-GFP-positive colonies per well in three independent experiments performed in triplicate (Figure 4B). No colonies were observed without Gcsf or after empty vector transfection. Expanded colonies showed no detectable genomic integration or expression of GY118F (Figure 4C). They exhibited the ground state marker profile consistent with conversion to iPSCs (Figure 4D). These observations indicate that a relatively short burst of Jak/Stat3 activation is sufficient in conjunction with 2i to elicit EpiSC reprogramming.

### Stat3 Acts Combinatorially with Klf4 or Nanog

Stat3 has multiple targets (Bourillot et al., 2009). These include Klf4, which is a known reprogramming factor for both EpiSCs (Guo et al., 2009; Hanna et al., 2009) and somatic cells (Takahashi and Yamanaka, 2006). However, Lif increases reprogramming efficiency of Klf4-transfected EpiSCs (Figure 1A) and Stat3 does not induce Klf4 in EpiSCs, suggesting that Klf4 is not the primary mediator of Jak/Stat3-induced reprogramming. We established EpiSCs expressing both Klf4 and GY118F transgenes. These cells generated Oct4-GFP colonies in 2i plus Gcsf at 10-fold higher frequency than cells expressing either construct alone (Figure 4E). We observed a similar combinatorial effect of combining Gcsf stimulation of GY118F with expression of Nanog. Marker profiling confirmed transcriptional resetting (Figure 4F). These findings indicate that the contribution of Stat3 to molecular reprogramming extends beyond any induction of *Klf4* or *Nanog*.

We then investigated whether Stat3 activation may prime EpiSCs for reprogramming. GY118F transfectants were exposed to Gcsf then transferred to 2i/Lif without Gcsf. An average of four colonies per plate was obtained after 48 hr of prestimulation (Table 2). No consistent effect with 24 hr pretreatment makes it unlikely that this result can be attributed simply to presence of active Stat3 at the time of transfer into 2i or persistence of Gcsf after media change. We repeated the experiment in cells doubly transfected with GY118F and Klf4 or Nanog. In both cases, 24 hr prior exposure to Gcsf substantially increased iPSC colony on transfer to 2i/Lif. This effect was more pronounced for 48 hr preincubation (Table 2). These data indicate that activation of Stat3 in EpiSCs acts to poise them for reprogramming.

### Increased Activation of Jak/Stat3 Contributes to Somatic Cell Reprogramming

**Figure 5:**
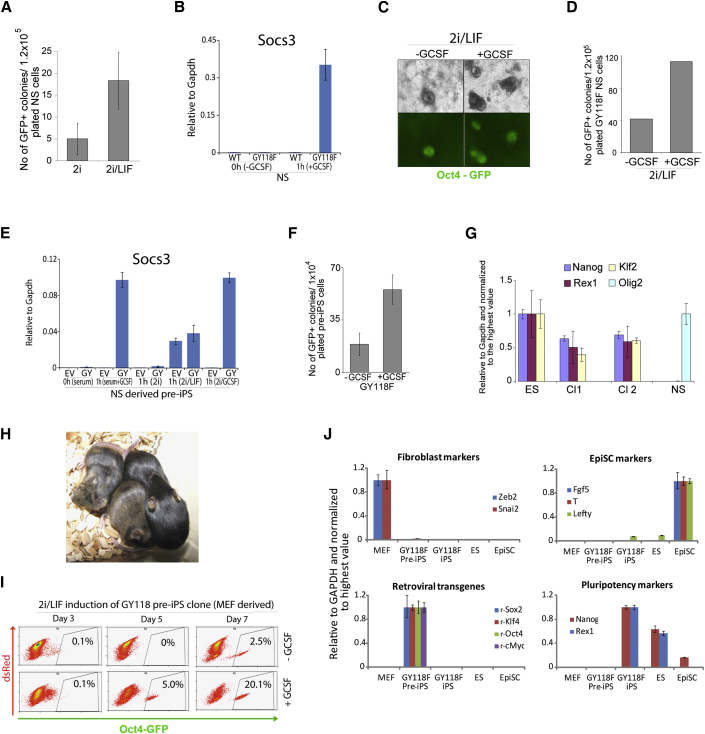
Activation of Jak/Stat3 Enhances Somatic Cell Reprogramming (A) Number of Oct4-GFP-positive iPSC colonies generated from NSCs transduced with Oct4, Klf4, and cMyc and cultured in 2i or 2i/Lif. (B) Induction of Socs3 in GY118F-transfected NSCs stimulated with Gcsf for 1 hr. (C) Representative images of iPSC colonies generated from GY118F NSCs transferred 5 days after retroviral transduction into 2i/Lif with or without Gcsf. (D) Number of Oct4-GFP-positive colonies obtained in (C). (E) Induction of Socs3 in GY118F (GY) NSCs derived from incompletely reprogrammed cells (pre-iPSCs) stimulated with Lif or GCSF for 1 hr. Empty vector (EV) transfectants were treated in parallel. (F) Number of Oct4-GFP-positive iPSC colonies from GY118F NSCs passaged at day 5 after reprogramming factor transduction and 2 days later transferred to 2i/Lif with or without GCSF. Empty vector NSC transfectants showed no response to Gcsf. (G) qRT-PCR analysis of the pluripotency markers Nanog, Rex1, and Klf2 and of the NSC marker Olig2 in two iPSC clones (cl) generated in the presence of Gcsf as in (F). (H) Two chimeras and nonchimeric littermates generated after injection of cl1 GY118F NS-iPSCs into C57BL/6 host blastocysts. (I) Frequency of Oct4-GFP-positive cells produced from fibroblast-derived pre-iPSCs transfected with GY118F and cultured in 2i/Lif with or without Gcsf. (J) Marker profile of iPSCs generated in Gcsf from GY118F-transfected fibroblast pre-iPSCs. Error bars in (B), (E), and (F) are standard deviations from the mean of triplicate determinations. In (G) and (J), error bars indicate the range of fold change relative to the sample with highest expression.

We investigated whether activation of Stat3 may also be limiting for somatic cell reprogramming. We first examined neural stem cells (NSCs) derived from adult mouse brain. If cultured in 2i/Lif, NSCs reprogram relatively rapidly and efficiently to chimera-competent iPSCs upon transduction with Oct4, Klf4, and cMyc retroviruses. (Silva et al., 2008). We compared the yield of Oct4-GFP-positive colonies obtained in 2i alone or in 2i plus Lif. In the presence of Lif, we observed a 3- to 4-fold increase in the number of colonies (Figure 5A). We then transfected O4G NSCs with GY118F. Gcsf induction of *Socs3* confirmed that the transgene was functional (Figure 5B). These cells were transduced with reprogramming factors and 5 days later transferred into 2i/Lif with or without Gcsf. In Gcsf, we observed a more than 2.5-fold increase in the number of Oct-GFP-positive colonies produced at 14 days (Figures 5C and 5D). We also passaged transduced NSCs at day 5 and maintained them in serum for a further 2 days as an incompletely reprogrammed population. Gcsf induced Socs3 to a higher level than Lif in these cells (Figure 5E). Upon exchange into 2i/Lif, addition of Gcsf yielded a 2.5- to 3-fold increase in the number of Oct4-GFP-positive colonies produced (Figure 5F). Gcsf had no effect on cells without GY118F. Two colonies from this experiment were expanded and analyzed by qRT-PCR (Figure 5G). They expressed ESC markers Nanog, Rex1, and Klf2, with loss of the NSC marker Olig2. We injected these cells into blastocysts and obtained viable chimeras (Figure 5H).

We also analyzed a stable clone of incompletely reprogrammed cells generated by retroviral transfection of O4G embryonic fibroblasts with Oct4, Sox2, Klf4, and cMyc and culture in the presence of serum. These Oct4-GFP-negative pre-iPSCs can progress to GFP-positive germline-competent iPSCs on transfer into 2i/Lif (Silva et al., 2009). We transfected pre-iPSCs with GY118F. Stable transfectants were changed into 2i/Lif with or without Gcsf. In Gcsf, Oct4-GFP reporter activity emerged earlier and the number of reprogrammed cells was increased by 8-fold at 7 days (Figure 5I). Pre-iPSCs exhibit a distinct marker profile from fibroblasts, EpiSCs, or ESCs and are characterized by continued expression of retroviral transgenes (Figure 5J). Oct4-GFP-expressing derivatives obtained by Gcsf treatment in 2i exhibited silencing of retroviral transgenes and upregulation of ground state ESC markers Nanog and Rex1 (Figure 5J).

## Discussion

By defining limiting components, it may be possible to achieve more efficient molecular reprogramming. The present findings reveal that insufficient activation of Jak/Stat3 restricts the acquisition of pluripotency. Therefore, Lif/Jak/Stat3 should be restored to the main stage among molecular regulators of pluripotent status. The results also highlight the importance of extrinsic stimuli in inhibiting or promoting cellular reprogramming.

We identified the contribution of Lif/Jak/Stat3 in the context of EpiSCs. This system is attractive because single factors are sufficient to induce reprogramming and the effect is exerted only after withdrawal of activin and Fgf (Guo et al., 2009). Recently, it has been reported that EpiSC populations cultured on feeders may spontaneously convert into ESCs (Bao et al., 2009; Greber et al., 2010). Our findings lend support to the speculation that Lif may drive this spontaneous process (Bao et al., 2009). Although in defined conditions stimulation of endogenous Lif receptor is not sufficient for reprogramming, chronic Lif stimulation may be effective in the presence of feeder- and serum-derived factors. It is noteworthy that EpiSCs cultured on feeders exhibit detectable expression of reprogramming factors Klf2 and Klf4 (Greber et al., 2010; Tesar et al., 2007), which are absent in EpiSCs cultured in feeder-free conditions with activin and Fgf2. Furthermore, levels of Nanog are low in defined conditions but are comparable to those in ESCs for EpiSCs on feeders. The presence of Klfs and Nanog may create a permissive context for reprogramming. Weak activation of Stat3 and lack of biological response to Lif are shared features of human embryo-derived stem cells and mouse EpiSCs (Brons et al., 2007; Daheron et al., 2004; Tesar et al., 2007; Thomson et al., 1998). It may therefore be of interest to engineer increased activation of Stat3 in human embryo-derived and induced pluripotent stem cells.

The relevance of EpiSC reprogramming to somatic cells may be questioned. However, as shown here for Lif/Stat3, all factors identified to date as mediators of EpiSC reprogramming are also effective in somatic cells. We note that iPSC colonies can be obtained without Lif, although at reduced frequency. This could be attributable to autocrine or paracrine expression of Lif. However, some reprogrammed colonies were obtained even in the presence of Jak inhibitor. This suggests that Stat3 may not be absolutely required for reprogramming. In ESCs, Lif is a potent stimulus of self-renewal but can be bypassed in certain circumstances (Ying et al., 2008). A facultative role is also apparent in the mouse blastocyst where the Lif/Stat3 pathway becomes essential only during the extended maintenance of epiblast in diapause (Nichols et al., 2001).

Even if not essential, activation of Jak/Stat3 appears to be limiting for induction of pluripotency. The effect of Jak/Stat3 is combinatorial with other reprogramming factors and is not mediated by induction of Klf4. This implies that one or more additional target(s) of Jak/Stat3 play a pivotal role. Identification of genes that are induced by Jak/Stat3 in EpiSCs may contribute to delineating the molecular building blocks of induced pluripotency and achieving a highly efficient deterministic reprogramming system.

## Experimental Procedures

### Cell Culture

EpiSCs were derived from E5.75 mouse embryos in N2B27 medium on fibronectin-coated wells (Guo et al., 2009) with activin (20 ng/ml) and Fgf2 (12 ng/ml) prepared in-house. ESCs and iPSCs were cultured in 2i/Lif comprising the Mek inhibitor PD0325901 (1 μM), GSK3 inhibitor CHIR99021 (3 μM), and leukemia inhibitory factor (Lif, 100 U/ml) in N2B27 medium (Ying et al., 2008). Cells were expanded by dissociation with accutase and passaged every 2–3 days. Gcsf (Preprotech) was used at 30 ng/ml or prepared in-house and titered against ESCs expressing GY118F (Burdon et al., 1999b). Jak inhibitor 1 (Calbiochem) was used at 0.6 μM, which inhibits ESC colony formation in Lif and serum (Chambers et al., 2003) but not in 2i. For colony forming assays, dissociated cells were plated at 600 cells per well in laminin-coated 6-well plates. Colonies were stained for alkaline phosphatase after 5 days (Sigma). Bright-field images were analyzed with an in-house macro in ImageJ and scored as wholly stained, mixed, or unstained (Hall et al., 2009).

### Electroporation and Transfection

For plasmid transfection, EpiSCs were electroporated with linearized pPyCAGGY118FiresZeo or pPyCAGSTAT3ER^T2^iresZeo. Stable transfectants were selected in 100 ng/ml zeocin (Invitrogen). Single colonies were picked and expanded. For transient expression, EpiSCs were transfected with Lipofectamine 2000 and after 24 hr, 2 × 10^4^ cells were plated per well into 6-well tissue culture plates. Cells were maintained in activin plus Fgf for 24 hr, then exchanged into 2i/Gcsf.

*PiggyBac* transfectants were established as described (Guo et al., 2009) with selection in 100 μg/ml hygromycin. For transgene excision, 5 × 10^5^ cells were treated with tatCre (Peitz et al., 2002) in N2B27. Five days later, DsRed-negative cells were purified by flow cytometry and plated at low density (600 cells/6-well). Nonfluorescent colonies were picked and expanded for characterization.

### Epi-iPSC Induction and Propagation

EpiSCs were plated at 2 × 10^4^ cells per well of 6-well tissue culture plates in activin plus Fgf. After 24 hr, medium was changed as specified and subsequently refreshed every other day. Numbers of Oct4-GFP- or Nanog-GFP-positive colonies were counted after 8 days. Fluorescent ESC-like colonies were individually picked after 10–12 days and expanded as clones, replating every 3–4 days. To test the contribution of Stat3 activation early in reprogramming, EpiSCs were plated as above and after 24 hr the medium was changed to 2i/Gcsf for 24 hr or 48 hr before rinsing in PBS and transfer to 2i/Lif. For pretreatment with Gcsf, EpiSCs were cultured in activin/Fgf2 with Gcsf for 24 hr or 48 hr before wash and transfer to 2i/Lif.

### Somatic Cell Reprogramming

Retroviral preparation was performed as described (Takahashi and Yamanaka, 2006; Silva et al., 2008). Adult brain-derived neural stem cells (NSCs) carrying the O4G reporter (Silva et al., 2008) were stably transfected with PB-GY118F or empty PB vector. For transduction with reprogramming factors, NSCs were plated overnight at 1.2 × 10^5^ per 10 cm^2^ well then incubated in virus/polybrene containing supernatants for 24 hr. Three days after transduction, cultures were changed from serum-free NSC medium into medium containing 10% FCS and Lif. Two days later they were transferred into 2i or were passaged and cultured for a further 2 days before changing to 2i. The O4G mouse embryo fibroblast-derived pre-iPSC clone was isolated as described (Silva et al., 2009) after retroviral transduction with Oct4, Klf4, Sox2, and cMyc. PB-GY118F was introduced by nucleofection (Amaxa) and transfectants selected in medium containing 10% FCS and Lif.

### Chimeras

Mouse studies were carried out in a designated facility under licenses issued by the United Kingdom Home Office. Chimeras were produced by microinjection into C57BL/6 blastocysts and detected by agouti coat coloring.

### PCR and QPCR

For qRT-PCR, total RNA was prepared with the RNeasy mini kit (QIAGEN) with DNaseI treatment. First strand cDNA was synthesized with SuperscriptIII reverse transcriptase (Invitrogen). Real-time PCR was performed with Taqman Gene Expression Assays (Applied Biosystems). Gene expression was determined relative to Gapdh via the ΔCt method. Primers and Taqman assays are listed in Supplemental Information.

### Immunoblotting

Cells were washed with cold PBS and harvested in 200 μl RIPA lysis buffer. Samples were sonicated and centrifuged at 13000 rpm for 15 min to prepare supernatant. Protein concentration was determined by BCA assay (Pierce, Thermo Scientific). Total protein (40 μg) was fractionated on 4%–12% Bis-Tris Novex Gel and electroblotted onto Nitrocelulose membranes, and the membranes were probed sequentially with anti-p-Stat3 (Cell signaling), anti-Stat3 (BD Biosciences), and anti-αTubulin (Abcam). Blots were incubated with horseradish peroxidase-coupled anti-rabbit IgG or anti-mouse IgG and developed with ECL plus (Amersham). Membranes were stripped between probing by incubation at 70°C for 30 min in 62.5 mM Tris (pH 6.8), 2% SDS.

### Immunofluorescence

Cells cultured on slides for 24 hr were fixed in 4% PFA, blocked, and permeabilized with 1% BSA and 3% serum in PBS with 0.3% Triton X-100. Slides were incubated with H3K27me3 (Upstate) or Oct4 (Santa Cruz) antibodies at 4°C overnight, then rinsed and incubated with Alex594-conjugated goat anti-rabbit IgG and Alex488-conjugated goat anti-mouse IgG (Invitrogen) and counterstained with DAPI.

## Figures and Tables

**Table 1:** Chimera Generation from Epi-iPSC Lines

Cell Lines	Embryos Transferred	Mice Born	Chimeras
EpiY118F-iPSC	70	19/70 (27.1%)	4/19 (21.1%)
EpiY118F(T)-iPSC	40	18/40 (45%)	12/18 (66.7%)
EpiPBY118-iPSC	74	34/70 (48.6%)	13/34 (38.2%)
EpiPBY118FCre-iPSC	95	31/95 (32.6)	23/31 (74.2%)
EpiSTAT3ER-iPSC	33	15/33 (45.4%)	6/15 (40%)

**Table 2:** Number of Oct4-GFP^+^ Colonies Obtained in 2iLif after GCSF Pretreatment

Transfection	No Gcsf	Gcsf 24 hr^a^	Gcsf 48 hr^b^
Vector only	0	0	0
GY118F only	0	0	4 ± 2
Klf4+vector	4 ± 2.6	1.3 ± 1.1	2.3 ± 2
Klf4+GY118F	19.3 ± 5	138.6 ± 9	696.6 ± 80
Nanog+vector	4.6 ± 2	3 ± 1.7	3.6 ± 2.8
Nanog+GY118F	11 ± 4	145.6 ± 28.3	262.6 ± 20.6

Data are mean ± SD from triplicate assays.

a EpiSCs were pretreated in Activin/Fgf2/Gcsf for 24 hr, then switched to 2iLif.

b EpiSCs were pretreated in Activin/Fgf2/Gcsf for 48 hr, then switched to 2iLif.

## Supplemental Information

Document S1. Supplemental Experimental Procedures and Three Figures
