# Supplementary material for: Stat3 Activation Is Limiting for Reprogramming to Ground State Pluripotency
Source: Cell Stem Cell. 2010 Sep 3;7(3):319–28. doi: 10.1016/j.stem.2010.06.022 (PMC3459098; doi:10.1016/j.stem.2010.06.022)
Supplement: Document S1. Supplemental Experimental Procedures and Three Figures [file mmc1.pdf]

Cell Stem Cell, Volume *7*

Supplemental Information

Stat3 Activation Is Limiting for Reprogramming

to Ground State Pluripotency

Jian Yang, Anouk L. van Oosten, Thorold W. Theunissen, Ge Guo, Jose C.R. Silva, and Austin Smith

INVENTORY OF SUPPLEMENTAL INFORMATION

Figure S1. *Tbx3* is not expressed in EpiSCs and is not induced by Lif signalling in either EpiSCs or ES cells

Additional information for Figure 1 relating to *Tbx3*.

Figure S2. Epi-iPS cell derivation from GY118F EpiSCs using Gcsf in serum free medium without 2i

Additional information for Figure 2.

Figure S3. Characterisation of tatCre excision of transgene in *PB*-GY118F Epi-iPS cells.

Additional information for Figure 2.

List of PCR Primers and qPCR Taqman Assays

Figure S1. *Tbx3* is not expressed in EpiSCs and is not induced by LIF in either EpiSCs or ES cells

A

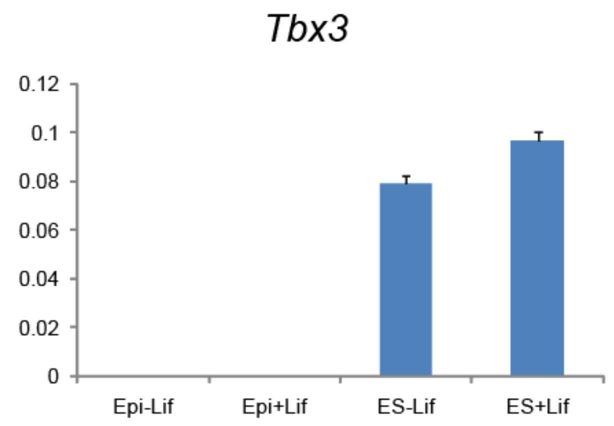

B

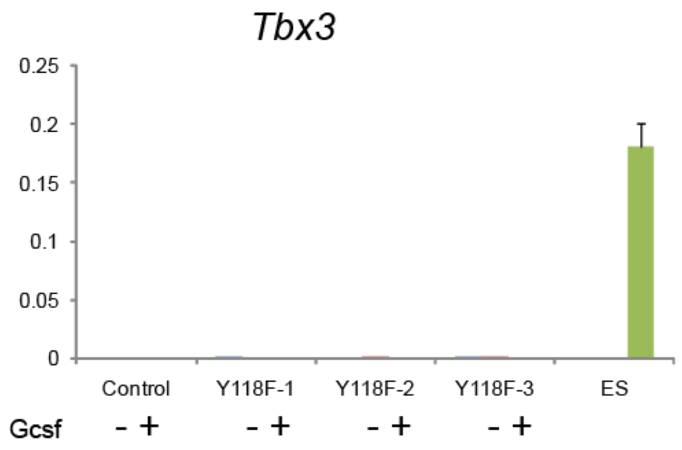

Taqman analyses were performed on total RNA preparations.

(A) EpiSCs or ES Cells were cultured in unsupplemented medium for 4 hours then stimulated with Lif for 1 hour.

(B) Empty vector or GY118F transfected EpiSCs maintained in Activin and Fgf2 were unstimulated or treated with Gcsf for 1 hour.

Error bars are standard deviations from the mean of triplicate determinations.

Figure S2 Epi-iPS cell derivation from GY118F EpiSCs using Gcsf in serum free medium without 2i

A

Numbers of Oct4GFP+ colonies per well after 8 days

Transgenic Lines	N2B27	N2B27/Gcsf
Vector	0	0
GY118F-1	0	8; 5; 10
GY118F-2	0	10; 3; 2
GY118F-3	0	15; 17; 22

B

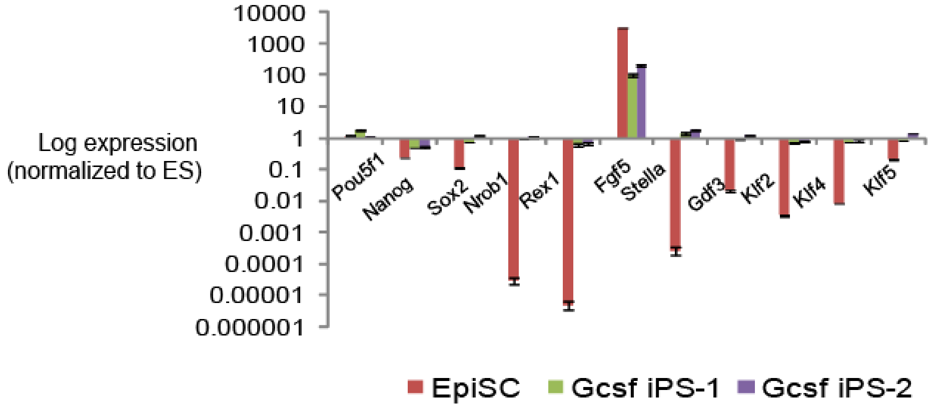

(A) Numbers of Oct4GFP positive colonies generated from three independent GY118F EpiSC clones by treatment with Gcsf in serum-free medium. *PB*-GY118F O4G EpiSCs were plated in triplicate at $2x10^4$ cells per well in Activin and Fgf2 for 24 hours then transferred into serum-free N2B27 medium supplemented with Gcsf for 8 days. Oct4-GFP positive colonies were then counted.

(B) qRT-PCR analysis of ground state and EpiSC markers. Oct4-GFP positive colonies were expanded in N2B27 plus Gcsf in the presence of puromycin (1μg/ml) to eliminate differentiated cells. Expression is relative to *Gapdh* and normalized to ES cells. The increased expression of *Fgf5* relative to ES cells in 2i is expected for cultures with active Fgf/Erk signaling.

Error bars are standard deviations from the mean of triplicate determinations.

Figure S3 Characterisation of tatCre excision of transgene in *PB*-GY118F Epi-iPS cells.

A

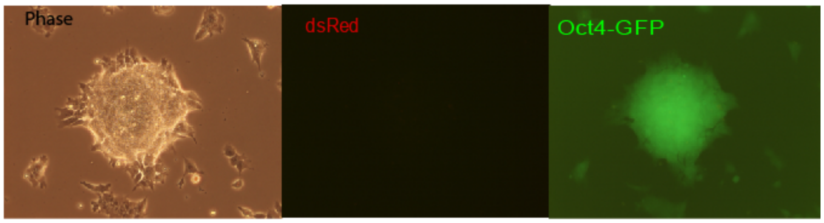

B

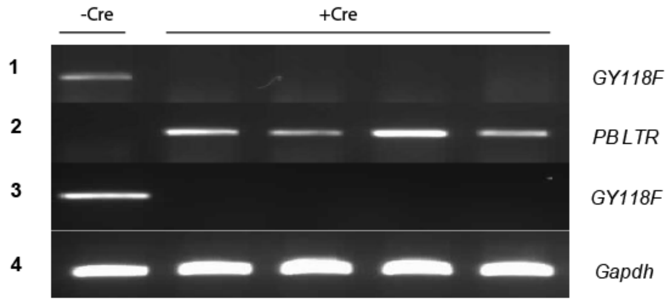

C

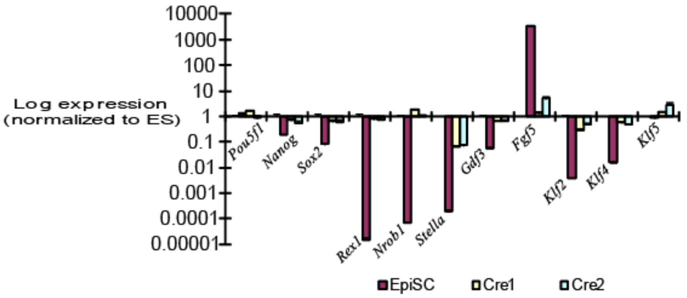

(A) Oct4 positive Epi-iPS colony obtained after tatCre protein transduction. Colony is dsRed negative, indicative of transgene deletion.

(B) Untreated and four tatCre-treated GY118F Epi-iPS clones. Genomic PCR analysis of transgene (panel 1, 2); RT-PCR analysis of transgene expression (panel 3); *Gapdh* expression as loading control (panel 4).

(C) qRT-PCR analysis of marker gene expression in Cre excised Epi-iPS cells. Gene expression level is relative to *Gapdh* and normalized to ES cells.

Error bars are standard deviations from the mean of triplicate determinations.

Supplemental Information on PCR and qPCR assays

List of primers

GY118F (gPCR and RT-PCR):

Forward cagcatgtctatgcctactc; Reverse accactgggcaatatgactc

PBLTR (gPCR)

Forward ccctagaaagataatcatattgtgac; Reverse ccctagaaagatagtctgcgtaaaat

STAT3ER (gPCR and RT-PCR):

Forward tagtgctgccccgtacctgaagac; Reverse cttcactgaagggtctggtaggat

Gapdh (RT-PCR):

Forward cccactaacatcaaatgggg; Reverse ccttccacaatgccaaagtt

Taqman assays

Il6st(gp130), Mm00439668_m1*; Lifr,* Mm00432942_m1*; Socs3,* Mm01249143_g1*; Stat3,* Mm01219775_m1*; Pou5f1 (Oct4)*, Mm00658129_gH; Sox2, Mm03053810_s1; *Klf4*, Mm00516104_m1; *Klf2*, Mm01244979_g1; *Klf5*, Mm00456521_m1; *Nanog*, Mm02384862_g1; *Rex1*,Mm03053975_g; *Fgf5*, Mm00438919_m1; *Gdf3,* Mm00433563_m1; *Nr0b1*, Mm00431729_m1; *Stella* (*Dppa3*), Mm00836373_g1; *T (brachyury),* Mm01318252_m1; *Lefty*, Mm00438615_m1; *Tbx3*, Mm00809779_s1; c-*myc*, Mm00487804_m1; *Snai2*, Mm00441531_m1; *Zeb2*, Mm00497193_m1*; Gapdh*, 4352339E

Expression of retroviral transgenes was determined using Custom TaqMan Gene Expression Assays (Applied Biosystems):

pMXs-Oct4 PF: 5'- TGGTACGGGAAATCACAAGTTTGTA, PR: 5'- GGTGAGAAGGCGAAGTCTGAAG, probe: 5'- FAM-CACCTTCCCCATGGCTG-MGB. pMXs-Klf4 PF: 5'-TGGTACGGGAAATCACAAGTTTGTA, PR: 5'- GAGCAGAGCGTCGCTGA, probe: 5'- FAM-CCCCTTCACCATGGCTG-MGB. pMXs-cMyc PF: 5'- TGGTACGGGAAATCACAAGTTTGTA, PR: 5'- GGTCATAGTTCCTGTTGGTGAAGTT, probe: 5'-FAM-CCCTTCACCATGCCCC-
